# Titanium Hypersensitivity as a Potential Contributor to Bronchopleural Fistula and Surgical Staple Extrusion Following Lung Cancer Surgery: A Case Report

**DOI:** 10.1155/crpu/1050765

**Published:** 2026-10-27

**Authors:** Mariana Lucena, Sara Regalo, Leonor Manso Preto, Ana Todo Bom, Frederico Regateiro

**Affiliations:** ^1^ Allergy and Clinical Immunology Department Coimbra University Hospitals Local Health Unit of Coimbra Coimbra Portugal huc.min-saude.pt; ^2^ Institute of Immunology Faculty of Medicine University of Coimbra Coimbra Portugal uc.pt; ^3^ Center for Innovative Biomedicine and Biotechnology (CIBB) Faculty of Medicine University of Coimbra Coimbra Portugal uc.pt; ^4^ UBIAir-Clinical and Experimental Lung Center and CICS-UBI Health Sciences Research Cente University of Beira Interior Covilhã Portugal ubi.pt

## Abstract

**Introduction:**

Titanium is widely regarded as a highly biocompatible material and is extensively used in surgical implants. Nevertheless, delayed hypersensitivity reactions are increasingly recognized and may act as potential contributing factors to implant‐related complications. Thoracic manifestations remain exceptionally rare, and their impact on clinical decision‐making is poorly described.

**Case Description:**

A 65‐year‐old man, with chronic obstructive pulmonary disease (COPD), a 60 pack‐year smoking history (former smoker since 2005) and severe gas transfer impairment, underwent video‐assisted thoracoscopic right lower lobectomy for lung adenocarcinoma. The postoperative course was complicated by a prolonged air leak requiring surgical staple reinforcement and talc pleurodesis. Approximately 2 months after surgery, the patient developed invasive pneumococcal disease with *Streptococcus pneumoniae* bacteremia, which resolved following appropriate antimicrobial therapy. Several months later, the patient developed a bronchopleural fistula (BPF) with endobronchial extrusion of titanium staples, confirmed by computed tomography and bronchoscopy. Given the unusual presentation, allergy evaluation was performed. Patch testing demonstrated epicutaneous sensitization with positive reactions to titanium (IV) oxalate hydrate, cobalt (II) chloride hexahydrate, and manganese chloride. The allergy work‐up had a direct therapeutic impact. Endoscopic closure with an Amplatzer device, a procedure documented for central BPFs, was initially considered but deliberately avoided because the considered device contains nitinol (a nickel–titanium alloy), which would expose the sensitized patient to additional titanium. Owing to the high surgical risk, conservative multidisciplinary management with close clinical and radiological surveillance was adopted.

**Discussion:**

BPF is a recognized complication after lung resection and is inherently multifactorial. In this patient, established surgical, anatomical (right lower lobectomy), and patient‐related comorbidity risk factors coexisted with documented titanium sensitization, suggesting that hypersensitivity may have acted as a potential contributing or modulating factor to impaired tissue integration and staple extrusion. This case highlights the diagnostic challenges of titanium hypersensitivity and the distinction between cutaneous sensitization and operative tissue allergy. Importantly, it illustrates how allergy assessment may directly influence therapeutic decision‐making by avoiding further exposure to potentially allergenic metallic devices. Titanium hypersensitivity should therefore be considered as an incremental factor in selected patients with unexplained implant‐related complications, even when conventional risk factors are present.

## 1. Introduction

Titanium and its alloys are widely used as biomaterials owing to their exceptional mechanical properties, corrosion resistance, and excellent biocompatibility [1–3]. As a result, titanium is extensively used in a broad range of medical specialties, including orthopedics, dentistry, neurosurgery, and cardiology [1, 2]. Despite its reputation as a biologically inert material, titanium hypersensitivity is increasingly recognized, although its true prevalence remains uncertain [1, 3]. Reported prevalence ranges from 0.2% to 3% in the general population, but many authors argue it is rarely detected rather than truly rare, often being overlooked due to nonspecific symptoms and a lack of standardized diagnostic tools [1]. Clinically, these delayed‐type IV immunopathological reactions may present as persistent pain, localized edema, erythema, eczema or systemic symptoms such as fatigue and chronic inflammation, eventually leading to implant failure or rejection [1, 3–5].

Diagnosing titanium hypersensitivity remains challenging. Cutaneous patch testing, the standard for metals like nickel, has limited reliability for titanium because titanium compounds do not easily penetrate the skin barrier [1, 2]. Although the lymphocyte transformation test (LTT) and its modification, MELISA, offer superior sensitivity by measuring systemic immune responses in vitro, their availability is limited and they lack uniform international standards [1, 2, 5].

In thoracic surgery, complications related to titanium implants are poorly characterized compared with other fields. Although titanium bars used in the Nuss procedure have been linked to allergic reactions, reports involving surgical staples are even scarcer [1]. Although mechanical staple‐line failure, leading to bronchopleural fistula (BPF), is a known risk (1.5%–2.0% rate), the role of metal hypersensitivity in these events is rarely considered [6].

This case highlights the potential contribution of titanium hypersensitivity to complex thoracic complications such as BPF and staple extrusion. Given the high mortality associated with BPF (up to 50%), recognizing individual biocompatibility issues is essential for optimizing therapeutic decisions and preventing recurrent implant‐related failures [6, 7].

## 2. Case Report

A 65‐year‐old man with a history of arterial hypertension, chronic obstructive pulmonary disease (COPD) and a 60 pack‐year smoking history (former smoker since 2005) presented with a right lower lobe adenocarcinoma. His baseline body mass index (BMI) was 22.39 kg/m^2^. Preoperative pulmonary function testing revealed an obstructive ventilatory defect with severe hyperinflation and no bronchodilator reversibility (FEV₁ 89.5% predicted, FVC 133% predicted, FEV₁/FVC 53%, TLC 140% predicted), along with a markedly reduced diffusing capacity for carbon monoxide (DLCO 6.4% predicted). In October 2023, he underwent video‐assisted thoracoscopic surgery (VATS), consisting of an atypical resection of a right lower lobe pulmonary nodule, right lower lobectomy, and mediastinal lymphadenectomy. During the postoperative hospital stay, he developed a prolonged air leak and underwent a second thoracoscopic procedure for closure of an alveolopleural fistula of the right upper lobe using surgical staples, followed by mechanical and chemical pleurodesis with talc poudrage.

Between December 2023 and March 2024, the patient received 4 cycles of adjuvant chemotherapy with carboplatin and vinorelbine. During the period from December 2023 to January 2024, he was hospitalized with invasive pneumococcal disease and multisensitive *Streptococcus pneumoniae* bacteremia. Diagnostic thoracentesis performed during this admission revealed an exudative pleural effusion. However, extensive microbiological evaluation of the pleural fluid, including Gram stain, aerobic and anaerobic bacterial cultures and mycobacterial studies, was completely negative. The bacteremia resolved following appropriate targeted antimicrobial therapy.

In March 2024, he presented with persistent cough, sputum production, and pleuritic chest pain. Chest computed tomography revealed a contained bronchopleural fistulous tract arising from the bronchial stump and extending into the pleural cavity with endobronchial extrusion of the surgical staples. The CT examination was performed at another institution, and the original images were not available to the authors for inclusion in this report. These findings were confirmed by bronchoscopy (Figure 1). Given the unusual clinical presentation, hypersensitivity to the surgical staple material was suspected and the patient was referred for urgent allergy evaluation. According to the manufacturer, the surgical staples were composed exclusively of titanium.

**Figure 1:**
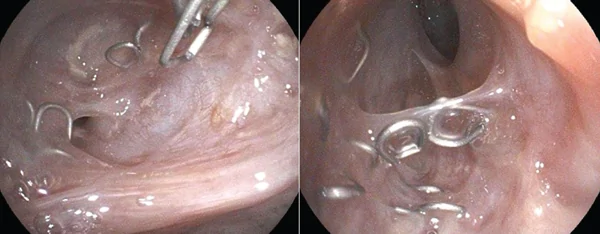
Bronchoscopic images of the bronchial stump showing surgical staples.

The patient was evaluated in the allergy department in June 2024. Patch testing was performed using the Chemotechnique Diagnostics 2024 Metal Series (MET‐1000) and Methacrylate Series (MA‐1000). As shown in Figure 2, patch testing demonstrated positive reactions to cobalt (II) chloride hexahydrate 1.0% pet (++), titanium (IV) oxalate hydrate 5.0% pet (+), and manganese chloride 2.0% pet (+). All other tested allergens yielded negative results.

**Figure 2:**
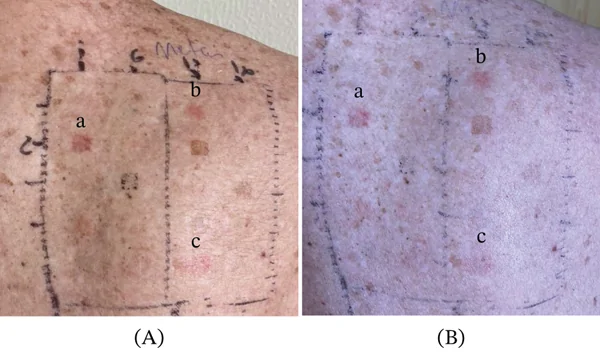
Patch test readings at (A) 48 h and (B) 72 h, showing positive reactions to (a) cobalt (II) chloride hexahydrate, (b) titanium (IV) oxalate hydrate, and (c) manganese chloride.

At follow‐up in July 2025, the patient remained clinically stable. The case was discussed within a multidisciplinary team and with the Department of Interventional Pulmonology. Endoscopic closure of the BPF using an Amplatzer device was considered. Although endobronchial deployment of Amplatzer occluder devices is documented in the literature as an effective, minimally invasive technique for closing central bronchial fistulae [8, 9]. This option was deemed unsuitable because the device contains nitinol (a nickel–titanium alloy). Placing a titanium‐containing nitinol structure would expose a sensitized patient to further immunological risk. Given the high risk associated with surgical reintervention, a conservative management strategy with close clinical and radiological surveillance was adopted and removal of the surgical material was deferred.

## 3. Discussion

### 3.1. Titanium Hypersensitivity and Implant‐Related Complications

The literature increasingly suggests an association between titanium hypersensitivity and implant‐related complications. Although titanium is chemically stable, metal ions and particles can be released due to mechanical stress or corrosion in inflamed environments [1, 2]. These particles act as haptens, binding to endogenous proteins to form an antigenic complex that triggers a T‐cell‐mediated Type IV hypersensitivity reaction [1, 2, 5]. In dentistry, hypersensitivity has been associated with spontaneous rapid implant exfoliation, gingival hyperplasia, and chronic mucosal pain [1, 2]. In orthopedics and neurosurgery, cases of impaired wound healing, persistent exanthema, and even massive cerebral edema following the placement of titanium clips or plates have been reported [1, 6]. Specifically, Gan et al. documented a foreign body granuloma caused by titanium clips, whereas other series show that symptoms consistently regress after the removal of the offending material [1, 6, 10]. The extrusion of a titanium hearing aid implant following necrotic ulceration further mirrors the potential for “rejection‐like” events in sensitized patients [1].

### 3.2. Diagnostic Challenges

One of the main controversies in the literature is the absence of a “gold standard” diagnostic test [1, 2]. Patch testing is frequently criticized for its low sensitivity [1, 5]. The choice of reagent is also critical. Although titanium dioxide often yields false negatives, titanium (IV) oxalate hydrate and titanium tetrachloride appear more reactive but can be unstable or cause skin irritation, complicating result interpretation [1, 2]. Conversely, many case reports rely on LTT or MELISA results to confirm sensitization, as these tests avoid the risk of iatrogenic sensitization and measure memory T‐cell proliferation [1, 2, 11]. Ultimately, most diagnoses are made retrospectively, based on the temporal association between implantation and symptoms, combined with complete resolution after explantation [2, 3].

### 3.3. Assessment of Causality in the Present Case

In this patient, the positive patch test to titanium (IV) oxalate hydrate provides objective evidence of sensitization, particularly as this specific salt is noted for being more effective in detecting titanium allergy than standard oxides [1, 2]. The endobronchial extrusion of the titanium staples is reminiscent of the “spontaneous exfoliation” seen in dental and ear implants, where inflammatory cascades prevent tissue integration and promote foreign body rejection [1].

However, causality must be analyzed alongside alternative explanations. BPF is a recognized, severe complication after lung resection and its pathogenesis is inherently multifactorial. In this patient, established surgical and host‐related risk factors coexisted with documented titanium sensitization. Anatomically and surgically, right lower lobectomy represents a major independent risk factor for BPF because of bronchial stump vascularity and mechanical stress [6, 7, 12]. Furthermore, the patient′s baseline comorbidity profile, specifically COPD, a heavy smoking history (60 pack‐years) and profound gas transfer impairment (DLCO 6.4%), significantly heightened his baseline vulnerability. Pre‐existing cardiothoracic and pulmonary comorbidities, particularly COPD and reduced ventilatory capacity, act as strong independent predictors of postoperative pulmonary complications [13]. This cumulative burden of chronic airway disease, combined with a prolonged postoperative air leak, mechanical/chemical pleurodesis, adjuvant chemotherapy and invasive pneumococcal bacteremia, created a severely compromised local and systemic environment for tissue healing.

In this patient, the positive patch test to titanium (IV) oxalate hydrate provides objective evidence of cutaneous sensitization. However, a clinical distinction must be drawn between epicutaneous sensitization and a proven operative local hypersensitivity reaction at the bronchial stump. Notably, bronchoscopy revealed no marked mucosal inflammation surrounding the exposed staples despite their endobronchial extrusion. Although the absence of intense mucosal inflammation does not rule out a delayed Type IV response, it indicates that titanium hypersensitivity should be interpreted as a potential contributing or modulating factor rather than the primary/sole cause of staple extrusion and fistula formation. The clinical outcome was most likely driven by the complex interaction between surgical‐anatomical risks, severe underlying lung disease, infectious insults, and immunological intolerance.

### 3.4. Clinical Implications

The allergy evaluation had a direct impact on clinical decision‐making. The decision to avoid the Amplatzer device was prudent, as these devices contain nitinol (a nickel–titanium alloy) [1]. Literature reports cases of severe generalized exanthema following the use of nitinol occluders in sensitized patients, requiring surgical extraction to resolve symptoms [1]. This case underscores the importance of considering metal hypersensitivity in patients with unexplained postoperative complications [1, 3]. Proactive screening, such as questioning patients about histories of jewelry allergy or prior implant intolerance, can prevent unnecessary morbidity [2, 4, 12]. In confirmed cases of hypersensitivity, alternative strategies should be employed, such as using absorbable sutures, polymer‐based clips or ceramic alternatives where applicable, to avoid repeated inflammatory insults [3, 4, 11].

## 4. Conclusion

This case illustrates that titanium hypersensitivity may represent a contributing factor in selected patients presenting with unexplained implant‐related complications. The diagnosis remains challenging because of the limited diagnostic utility of standard patch testing and the absence of a universally accepted gold standard [1, 2]. The clinical course was most likely multifactorial, involving anatomical risk factors, previous postoperative complications and possible immunological sensitization to titanium. The key learning point is that titanium hypersensitivity should be considered in the differential diagnosis of unexplained implant failures or persistent postoperative complications, particularly when the clinical course is not fully explained by more common causes, such as infection or technical error, whether these have been excluded or are present concurrently. Early recognition allows for informed therapeutic adjustments and the avoidance of further exposure to allergenic metallic materials [1, 3, 8].

## Funding

No funding was received for this manuscript.

## Consent

Written informed consent for publication of this case report and the accompanying images was obtained from the patient.

## Conflicts of Interest

The authors declare no conflicts of interest.

## Data Availability

The data that support the findings of this study are available on request from the corresponding author. The data are not publicly available due to privacy or ethical restrictions.
